# The surface area effect: How the intermediate dose spill depends on the PTV surface area in SRS

**DOI:** 10.1002/acm2.13203

**Published:** 2021-02-17

**Authors:** Dharmin D. Desai, E. L. Johnson, Ivan L. Cordrey

**Affiliations:** ^1^ Radiation Oncology CHI Memorial Hospital Chattanooga TN USA; ^2^ Department of Radiation Medicine University of Kentucky Chandler Medical Center Lexington KY USA

**Keywords:** brain, intermediate dose spill, PTV surface area, R50%, R50% prediction, SRS

## Abstract

**Purpose:**

Stereotactic radiosurgery (SRS) is rapidly becoming the standard of care for many intracranial targets. The characteristics of the planning target volume (PTV) can affect the intermediate dose spill and thus normal brain volume dose which is correlated with brain toxicity. R50% (volume receiving 50% of prescription dose divided by PTV volume) is a useful metric to quantify the intermediate dose spill. We propose a novel understanding of how the PTV surface area (SA_PTV_) affects the intermediate dose spill of SRS treatments.

**Methods:**

Using a phantom model provided by a computed tomography (CT) of the IROC Head Phantom® and Eclipse® Treatment Planning System, we investigate the relationship of R50% and SA_PTV_ in single‐target SRS treatments. The planning studies are conducted for SRS treatments on a Varian TrueBeam® linear accelerator with high‐definition MLC and a 6 MVFFF beam mode. These data are analyzed to ascertain trends in R50% related to SA_PTV_. Since SA_PTV_ is not available as a structure property in the Eclipse RTPS, we introduce an Eclipse script to extract PTV surface area of arbitrary‐shaped PTVs. We compare a physically reasonable theoretical prediction of R50%, R50%_Analytic_, to the R50% achieved in treatment planning studies.

**Results:**

The SRS phantom study indicates good correlation between the plan R50% and SA_PTV_. A near‐linear relationship of plan R50% vs SA_PTV_ is observed as predicted by the R50%_Analytic_ model. Agreement between plan R50% values and R50%_Analytic_ predictions is good for all but the very smallest PTV volumes.

**Conclusions:**

We demonstrate dependence of the intermediate dose spill measured by R50% on the SA_PTV_. We call that dependence the surface area effect. This dependence is explicit in the R50%_Analytic_ prediction model. The predicted value of R50%_Analytic_ for a given PTV could be used for guidance during SRS treatment plan optimization, and plan evaluation for that PTV.

## 1 INTRODUCTION

Stereotactic radiosurgery (SRS) and stereotactic radiotherapy (SRT) both refer to the highly conformal delivery of a very high dose, with very high spatial precision, to a target typically in the cranium. Stereotactic radiosurgery is a term reserved for a single fraction delivery while SRT can be three to five fractions. SRS/SRT is becoming standard of care for a host of small planning target volumes (PTVs) within the cranium.^1^, ^2^ Stereotactic radiosurgery/SRT is delivered on a variety of machine types including Gamma Knife (Elekta Instrument AB, Stockholm, Sweden), CyberKnife (Accuray, Sunnyvale, California), TomoTherapy (Accuray, Sunnyvale, California), and conventional C‐arm linear accelerators (linac) such as the Varian TrueBeam STx® with 120 leaf HD MLC or Edge radiosurgery system (Varian Medical Systems, Palo Alto, CA) and Elekta Versa HD (Elekta Instrument AB, Stockholm, Sweden). Photon energies of 6 MV and ^60^Co are commonly used. Current technology makes linac‐delivered SRS/SRT a good clinical option using both volumetric modulated arc therapy (VMAT) and dynamic conformal arc therapy (DCAT) techniques.^1^, ^2^

An important objective of SRS treatment planning and delivery is to minimize the nontarget brain dose by tightly conforming the prescription (Rx) dose to the target lesion with steep dose fall‐off outside the target surface, with the goal of minimizing the intermediate dose spill. The degree to which normal brain tissue is irradiated in SRS is known to be associated with complications such as radiation necrosis.^3^ Various SRS studies have evaluated the effect of dose delivered to normal brain tissue in the dose fall‐off region on the development of complications from radionecrosis.^3^, ^4^ For example, Flickinger et al.^3^ developed a predictive model for symptomatic postradiosurgery brain injury (necrosis) when treating arteriovenous malformations using SRS techniques based in part on the parameter V12 Gy (brain Volume receiving 12 Gy or more, a volume dose statistic). A similar study was conducted by Minniti and co‐workers^5^ when treating brain metastases using SRS. That study showed evaluated risk of developing radionecrosis associated with brain volume‐specific doses between 10 and 16 Gy (i.e., V10–V16 Gy). These and other studies have shown that minimizing intermediate dose spill in SRS planning is an important goal when minimizing the risk of complications due to brain radionecrosis.

Various metrics have been devised to assess the level of dose fall‐off, or intermediate dose spill, in radiotherapy planning. In stereotactic body radiation therapy (SBRT), it is common to use the metric R50%, defined as the ratio of the 50% Rx isodose cloud volume (V_IDC50%_) to the volume of the PTV (V_PTV_).^6^ The metric GI is commonly used in SRS planning when evaluating competing plans. Paddick^7^ defines the GI as the ratio of the 50% Rx isodose cloud volume (V_IDC50%_) to the 100%Rx isodose cloud volume (V_IDC100%_). This is essentially the form of GI as written by Zhao et al.^1^ Clearly, if the plan is perfectly conformal, V_IDC100%_ is equivalent to and spatially coincident with the PTV volume (V_PTV_) and GI is equivalent to R50%. But if V_IDC100%_ is not perfectly conformal to the PTV, plan flaws can be masked. For example, a V_IDC100%_ larger than V_PTV_ is possible and, in such a case, the GI would not adequately account for the normal tissue that falls within V_IDC100%_ but is outside the PTV surface. As a consequence, a plan with an acceptable GI could be an inferior plan in terms of the normal tissue outside of the PTV being radiated to a high dose. In a study of linac‐based RapidArc^®^ (Varian Medical Systems, Palo Alto, CA) SRS plans, Liu and co‐workers^2^ identified such a phenomenon where the RapidArc plans appeared to have noticeably large GI values than GammaKnife plans for the same patients. This was noted by Liu et al in the statement “larger GI values for the RapidArc SRS plans are not because they have a larger 50% prescription isodose volume but because they all have smaller 100% prescription isodose volume,”^2^ that is, the RapidArc plans are more conformal than the GammaKnife plans. Another useful metric for quantifying intermediate dose spill in highly conformal treatment approaches is R50%.^6^ R50% is defined as the ratio of V_IDC50%_ to V_PTV_. While neither GI nor R50% are exactly the same as volume dose specifications such as V12 Gy, they can be considered reasonable surrogates for volume dose values in plan optimization because of the nested nature of isodose lines. Considering that the standard Conformity Index (CI)^8^ is V_IDC100%_/V_PTV_, the R50% can viewed as the direct analog of CI for the IDC50% (R50% = V_IDC50%_/V_PTV_). In this study, we choose to use R50% as the intermediate dose spill metric for the following reasons. First, R50% removes the potential counterintuitive results of GI as described above that can mischaracterize the normal brain dose. And second, R50% is directly tied to the intermediate dose spill and the PTV volume regardless of the actual dose gradient and thus better indexes the normal brain volume dose.

There are many factors that may influence the intermediate dose spill including PTV location, PTV volume, beam geometry, delivery method, and the distribution of critical structures. In this work, we focus on a limited number of factors, namely V_PTV_ and PTV surface area (SA_PTV_). It is common in the literature for authors to organize treatment planning outcomes as a dependence on V_PTV_ as this parameter is readily available in the radiation treatment planning system (RTPS) contour statistics.^4^, ^5^, ^7^, ^9^, ^10^, ^11^ Examples include optimal Isodose Rx line or V12 Gy vs V_PTV_. One characteristic that is typically observed in these published studies is a dispersion of results for a cohort of PTVs with similar V_PTV_. At least one study attempted to reconcile the nature of this observed dispersion by considering the PTV shape and surface area but were unable to incorporate shape in any effective manner.^10^ We believe the SA_PTV_ (i.e., a shape dependency) plays an important role in achievable intermediate dose spill metrics such as R50% in highly conformal approaches such as SRS and we have conducted a study to test this hypothesis. This work explores SA_PTV_ as was done previously in our study of R50% in SBRT,^12^ but here we examine R50% in cranial SRS. This work also builds on our previous efforts which derived a semi‐empirical equation for an approximation of R50%, which we refer to as R50%_Analytic_, based on V_PTV_ and SA_PTV_ in lung SBRT.^13^ The R50%_Analytic_ value is considered to be a prediction of the R50% result that may be achieved in highly conformal treatment techniques given SA_PTV_ and V_PTV_ for the treated PTV. Utilizing an anthropomorphic head phantom study, we have applied the R50%_Analytic_ approach devised in the lung SBRT study to cranial SRS. We demonstrate that the dependence of the achievable R50% on the SA_PTV_ is consistent with previous lung SBRT results.^12^ We test the R50%_Analytic_ methodology on single cranial PTVs of various shapes and sizes. Plans have been optimized to achieve minimum R50% values for delivery utilizing MLC, linac‐based SRS techniques. This scope is believed to be representative of typical, clinical cranial SRS cases.

## 2 MATERIALS AND METHODS

### 2.A Phantom model

**FIG. 1:**
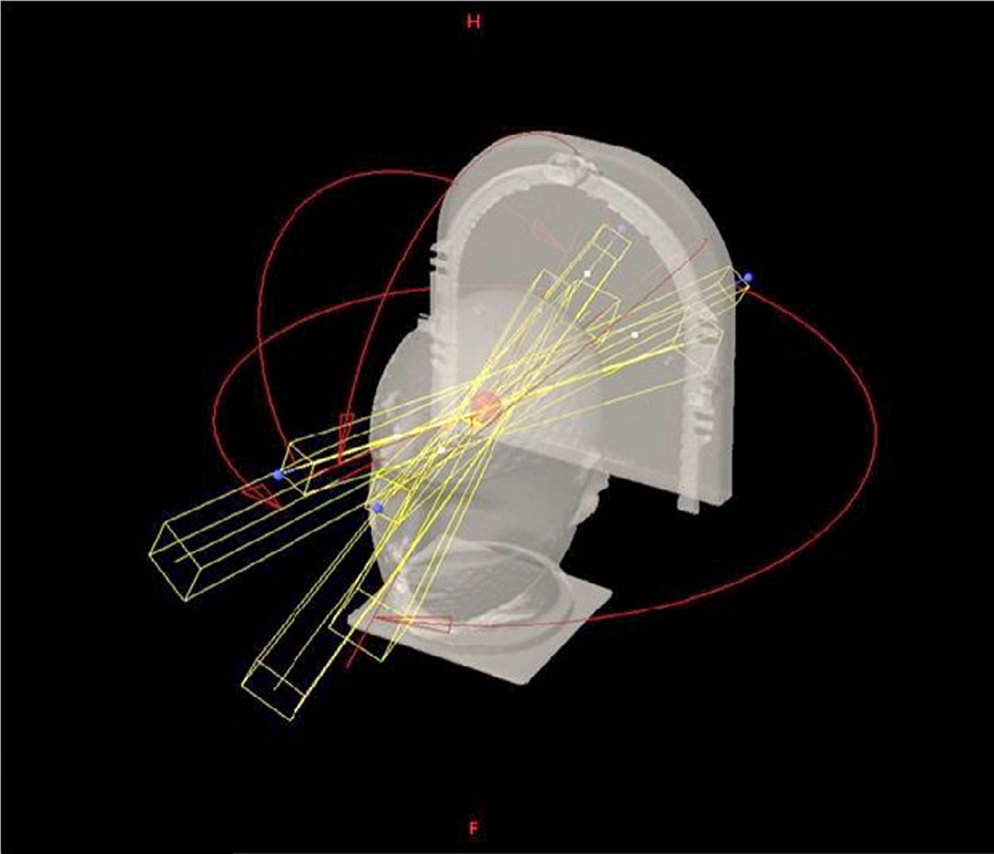
The five hemi‐arcs beam arrangement used for treatment planning in the Δr and SRS PTV studies.

A high‐resolution treatment planning CT of the IROC Head Phantom® (IROC Houston QA Center, Houston, TX) was utilized to acquire the anthropomorphic head phantom model. Images were obtained on a Philips Big Bore Brilliance CT Simulator (Philips Healthcare North America, Andover MA). Images were acquired on a 512 × 512 matrix using a 36 cm reconstruction field of view yielding pixel dimensions 0.07 × 0.07 cm. A helical acquisition with a 1‐mm slice spacing was utilized to acquire a CT study comprised of 323 images. A surface rendering of the model can be seen in Fig. 1.

### 2.B Treatment planning optimization and dose calculation methodology

All treatment planning was performed on a Varian Eclipse Radiation Treatment Planning System (RTPS) v15.6 (Varian Medical Systems Palo Alto CA). Image segmentation was performed using the Contouring module in Eclipse. For the purpose of this study, we ignore the IROC PTV and create unique sets of centrally located PTVs in the cranial cavity with well‐controlled characteristics. Plans were created for delivery on a Varian TrueBeam STx® linac, 6XFFF mode, and having a 120 leaf HD MLC. Treatment delivery utilizes a RapidArc^®^ VMAT approach. Beam geometry consisted of five hemi‐arcs each spanning 150 arc degrees. To maximize the degree of non‐coplanar beam delivery, each hemi‐arc used a unique couch angle including 355°, 315°, 270°, 45°, and 5°. The geometric configuration of gantry, collimator, couch, and phantom is shown Fig. 1. This geometry is both clinically reasonable and highly conformal for a central cranial tumor treated on a conventional C‐arm linac because it utilizes a nearly full 2π solid angle beam entry geometry and no beam line overlaps with another beam line other than when close to the target.

A fixed SRS Rx dose of 18 Gy in one fraction was used for each plan with the requirement that 99% of the PTV volume receive the Rx dose (i.e., D99% Rx condition).^14^ The Eclipse automatic Normal Tissue Objective (NTO) as well as standard dose‐limiting shells were utilized in the optimization to minimize dose gradient and encourage high‐dose conformality.^11^, ^15^, ^16^ Each plan was optimized with the same set of criteria seeking a minimum R50% and high‐dose conformality. Conformality was assessed using the standard Conformity Index (CI) defined as the ratio of the prescription dose volume to the PTV volume.^8^ The Eclipse algorithm PO v15.6 was used for all optimizations. Eclipse AAA convolution v15.6 was used for the final dose distribution calculation implemented on a 1‐mm grid point spacing matrix. R50% values so obtained were compared to SA_PTV_ and the prediction given by the R50%_Analytic_ methodology.

### 2.C R50%_Analytic_ methodology and the dependence on PTV surface area

It is common in the literature for authors to organize treatment planning outcomes as a dependence on V_PTV_ as this parameter is readily available in the RTPS structure statistics.^4^, ^5^, ^7^, ^9^, ^10^, ^11^ PTV shape and therefore the SA_PTV_ are typically unknown or not reported. Within these reported results, for a cohort of PTVs of given, nominal PTV volume, one can often observe dispersion in obtained plan metrics such as R50% which we surmise is an indication of the effect of the PTV surface area or shape on the observed outcome. The R50%_Analytic_ methodology developed in our previous work in lung SBRT^13^ attempts to incorporate the effects of PTV shape through the PTV surface area. As the details are available elsewhere, we only reproduce the final result for the form of R50%_Analytic_ as follows:

(1) $$R50{\%}_{\mathrm{Analytic}}=1+\frac{SA_{\mathrm{PTV}}}{V_{\mathrm{PTV}}}\Delta r\left\{1+\left[\frac{\Delta r}{r_{\mathrm{PTV}}}\right]+\frac{1}{3}{\left[\frac{\Delta r}{r_{\mathrm{PTV}}}\right]}^{2}\right\}$$

where

(2) $$r_{\mathrm{PTV}}={\left(\frac{3V_{\mathrm{PTV}}}{4\pi }\right)}^{1/3}$$

and $r_{\mathrm{PTV}}$ is the radius for an effective spherical shape for the given PTV volume. The ∆r is the dose drop‐off parameter and is addressed later in Section 2.D. The R50%_Analytic_ value obtained from Eq. (1) is understood to be a prediction of the approximate R50% obtainable in a treatment planning scenario for a PTV with known volume and surface area.

This result is obtained from a uniform expansion from the PTV surface by some distance Δr to where the dose drops to 50% of the Rx value. This ∆r is not explicitly obtainable from the derivation and cannot be calculated from first principles at this time, but is considered a parameter that is dependent on the treatment modality and must be measured directly. Values for Δr would be expected to depend on conditions such as photon energy, beam collimation, and the spatial distribution of beam lines. Given the likely dependence on the treatment delivery technology, we choose to measure Δr values for our model from a study of centrally located, spherical PTVs as described in Section 2.D.

To validate the effectiveness of Eq. (1) to predict the dependence of R50% on SA_PTV_, we create a series of PTVs all having the nominal volume 4 cm^3^. This removes any implied dependence on V_PTV_ in Eq. (1). One sphere and four cylinders are manually contoured in Eclipse with characteristics as indicated in Table 1. These PTVs of increasing SA_PTV_ are subjected to the treatment planning methodology as outlined in Section 2.B to minimize R50%.

**TABLE 1:** Nominal 4 cm^3^ isovolume PTVs used for the surface area dependence study. Shape characteristics are given as well as the calculated SA_PTV_. Treatment Planning R50% values are shown in the last column.

Description	Eclipse measured V_PTV_ (cm^3^)	Diameter (cm)	Height (cm)	SA_PTV_ (cm^2^)	Plan R50%
“Coin‐like” Cylinder	4.07	3.0	0.6	18.9	4.53
“Pencil‐like” Cylinder	4.00	1.0	5.1	17.6	4.28
“Shorter” Cylinder	4.00	1.4	2.6	14.5	3.56
“Minimum SA” Cylinder	3.96	1.7	1.7	13.8	3.40
Sphere	3.96	1.96	NA	12.1	2.95

### 2.D **Phantom study for the determination of** Δr

Nine spherical PTVs were manually contoured in Eclipse and located in the center of IROC Head Phantom® model described in Section 2.A. PTV volumes ranged from 0.19 to 44 cm^3^ and their characteristics are summarized in Table 2. Treatment planning was performed as outlined in Section 2.B. This scenario is expected to yield near‐minimum values achievable for Δr within our treatment conditions. Given the near‐2π distribution of beam directions using this delivery geometry, we expect isodose surfaces to be essentially spherical. Since the PTVs are also spheres, Δr would be readily obtainable from the difference of the radii of the 50%Rx isodose cloud (*r_SphVIDC50%_*) and the PTV (*r_SphPTV_*). The Eclipse RTPS provides a useful tool for this purpose known as the Gradient Measure (GM). In general terms, GM^9^ is defined for any shape 50%Rx isodose cloud and 100%Rx isodose cloud as follows:

(3) $$GM=r_{EqSphVIDC50\%}‐r_{EqSphVIDC100\%}$$

where $r_{EqSphVIDC50\%}$ and $r_{EqSphVIDC100\%}$ are the radii of spheres that are equal in volume to the actual V_IDC50%_ and V_IDC100%_, respectively. Given the uncomplicated conditions (i.e., centrally located PTVs and no normal structure constraints) for the optimization of these nine test spherical PTVs, we expect the dose distribution to be highly conformal and assume the 100%Rx isodose cloud would be very nearly coincident with the PTV volume, that is, have high conformality. The Conformity Index metric (CI) is used to prove all plans have an acceptable degree of conformality.^8^, ^17^ CI values < 1.1 are considered adequate to assure the required level of conformality. Therefore, it follows that Δr is well‐approximated by:

(4) $$\Delta r\;≅\;GM\;≅\;r_{Sph50\%RxIsodose}‐r_{\mathrm{SphPTV}}$$

**TABLE 2:** Spherical PTVs utilized in a phantom study to estimate Δr. Also shown are the CI values obtained indicating treatment planning achieved a high degree of conformality. Gradient Measure (GM) values shown are assumed to be equivalent to Δr as indicated in Eq. (4).

PTV volume (cm^3^)	Plan achieved CI	Eclipse GM (cm)
0.19	1.18	0.20
0.55	0.99	0.25
0.99	1.04	0.27
1.96	1.04	0.30
2.96	1.03	0.34
3.97	1.04	0.35
6.93	0.99	0.40
20.45	0.99	0.52
43.99	0.99	0.65

GM values reported by the Eclipse RTPS were used to obtain realistic estimates for Δr to be utilized in Eq. (1).

### 2.E Surface area script

At present we are not aware of a commercially available RTPS that reports the surface area of a segmented structure. However, it is possible to estimate the surface area of a 3D object utilizing the information contained in the 3D surface mesh representation. Using the 3D mesh geometry available in the Eclipse Scripting API, we developed a script to determine the surface area of contoured structures. This surface area script was validated on 23 spheres and cylinders for which the surface area could be calculated analytically for volumes ranging from 0.2 to 163 cm^3^. Once validated, the script was utilized to obtain the surface area estimates of the irregular PTVs used in Section 2.F which cannot be easily determined using analytical means.

### 2.F Phantom study for analysis of R50% obtained from clinically relevant SRS PTV volumes

A set of 20 PTVs of varying dimensions and shapes were created, including six spheres, six cylinders, and eight irregular shapes designed to mimic clinical PTVs. These PTVs were manually created using standard Eclipse contouring tools. The irregular shapes were created by starting with spheres and randomly creating indentations in the PTV surface using the eraser tool in Eclipse. The focus of the irregularly shaped PTVs was to create volumes concentrated in the smaller range of PTV sizes more typical of SRS lesions. Treatment planning methods described in Section 2.B are used to obtain R50% values for these volumes. Corresponding R50%_Analytic_ values are also determined using Eq. (1) with Δr estimated from a power law fit to the data obtained from the study described in Section 2.D. The PTV characteristics utilized are summarized in Table 3.

**TABLE 3:** Characteristics of PTVs used in the SRS R50% phantom study. Also shown are the CI and R50% obtained from treatment planning and the R50%_Analytic_ result. The R50%_Analytic_ value is in good agreement with planned R50% obtained as indicated in the last column.

PTV shape	Volume (cm^3^)	Surface area (cm^2^)	Plan achieved CI	Plan achieved R50%	R50%_Analytic_	Plan R50%/R50%_Analytic_ Ratio
Sphere	0.36	2.40	1.10	3.73	3.42	1.09
	1.21	5.45	1.04	3.04	2.85	1.07
	2.96	9.93	1.01	2.64	2.58	1.02
	9.83	22.12	1.00	2.39	2.34	1.02
	23.19	39.25	0.99	2.22	2.23	1.00
	43.99	60.42	0.99	2.17	2.17	1.00
Cylinder	0.38	2.85	1.22	4.70	3.73	1.26
	1.26	6.42	1.20	3.72	3.10	1.20
	3.01	11.57	1.10	2.80	2.81	1.00
	9.98	25.91	1.05	2.57	2.55	1.01
	23.70	45.86	1.05	2.52	2.41	1.05
	44.38	69.77	1.04	2.16	2.34	0.92
Irregular	0.18	1.57	1.18	4.60	4.06	1.13
	0.60	3.70	1.03	3.35	3.34	1.00
	1.00	5.08	1.10	3.16	3.03	1.04
	1.90	7.96	1.02	2.88	2.83	1.02
	2.95	10.66	1.07	2.82	2.70	1.04
	3.99	13.33	1.00	2.64	2.66	0.99
	7.05	20.86	1.00	2.55	2.64	0.97
	20.09	43.80	1.01	2.35	2.53	0.93
Mean(sd) Plan R50%/R50%_Analytic_ ratio						1.04 (0.08)

## 3 RESULTS

### 3.A **Phantom study for the determination of** Δr

**FIG. 2:**
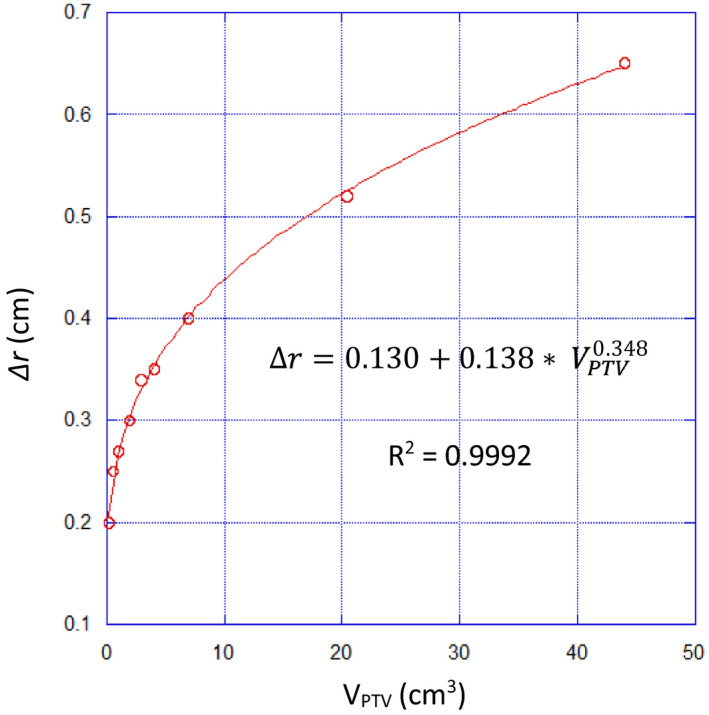
Results of the spherical PTV phantom study to determine the dependence of Δr on V_PTV_ for the treatment planning conditions described in Section 2.B. Also shown is the result of a power law fit to these data.

Estimates of Δr obtained in the phantom study are summarized in Table 2 and Fig. 2. Plans used to evaluate Δr resulted in mean(sd) CI values of 1.03(0.06) indicating acceptable conformality to the PTV. The largest outlier, CI = 1.18, occurs for the smallest volume, 0.19 cm^3^, and is likely due to voxelation and discretization artifacts for very small volumes. A power law fit to these data shows good correlation (R^2^ = 0.9992):

(5) $$\Delta r=0.130+0.138{\left(V_{\mathrm{PTV}}\right)}^{0.348}$$

The values obtained from Eq. (5) are used as input to Eq. (1) to obtain R50%_Analytic_ predictions.

### 3.B R50%_Analytic_ methodology and the dependence on PTV surface area

**FIG. 3:**
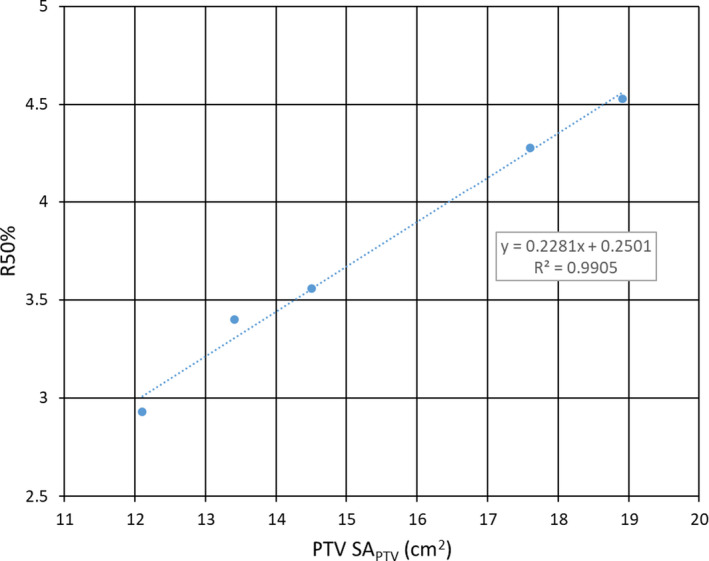
Plot of R50% vs PTV surface area (SA_PTV_) for the 4 cm^3^ PTVs described in Table 1. Notice the linear dependence of the R50% values on SA_PTV_ as suggested by Eq. (1).

Figure 3 and Table 1 summarize the results obtained from the treatment planning study on the 4 cm^3^ nominal volume PTVs each having a different surface area. Plan R50% values show a clear linear dependence on SA_PTV_ that is well‐correlated (R^2^ = 0.9905). Our claim that Eq. (1) has predictive power in SRS treatment planning outcomes for R50% is supported by these results.

### 3.C Surface area script

Validation of the surface area script for the 23 spheres and cylinders tested showed good agreement with the known surface area. The average percent difference between the script surface area and the analytically calculated surface area was 0.96% ± 0.92%. The surface area script was within a few percent for every structure tested. The deviation between analytically calculated and script surface area was largest for very small volumes where the voxelation distortions of the contours become significant, especially at the axial planes defining the longitudinal bounds of the structures where the volume is extended one‐half slice thickness in the longitudinal direction. This script was used to obtain the SA_PTV_ of all PTVs reported in Section 3.D.

### 3.D Phantom study for analysis of R50% obtained from clinically relevant SRS PTV volumes

**FIG. 4:**
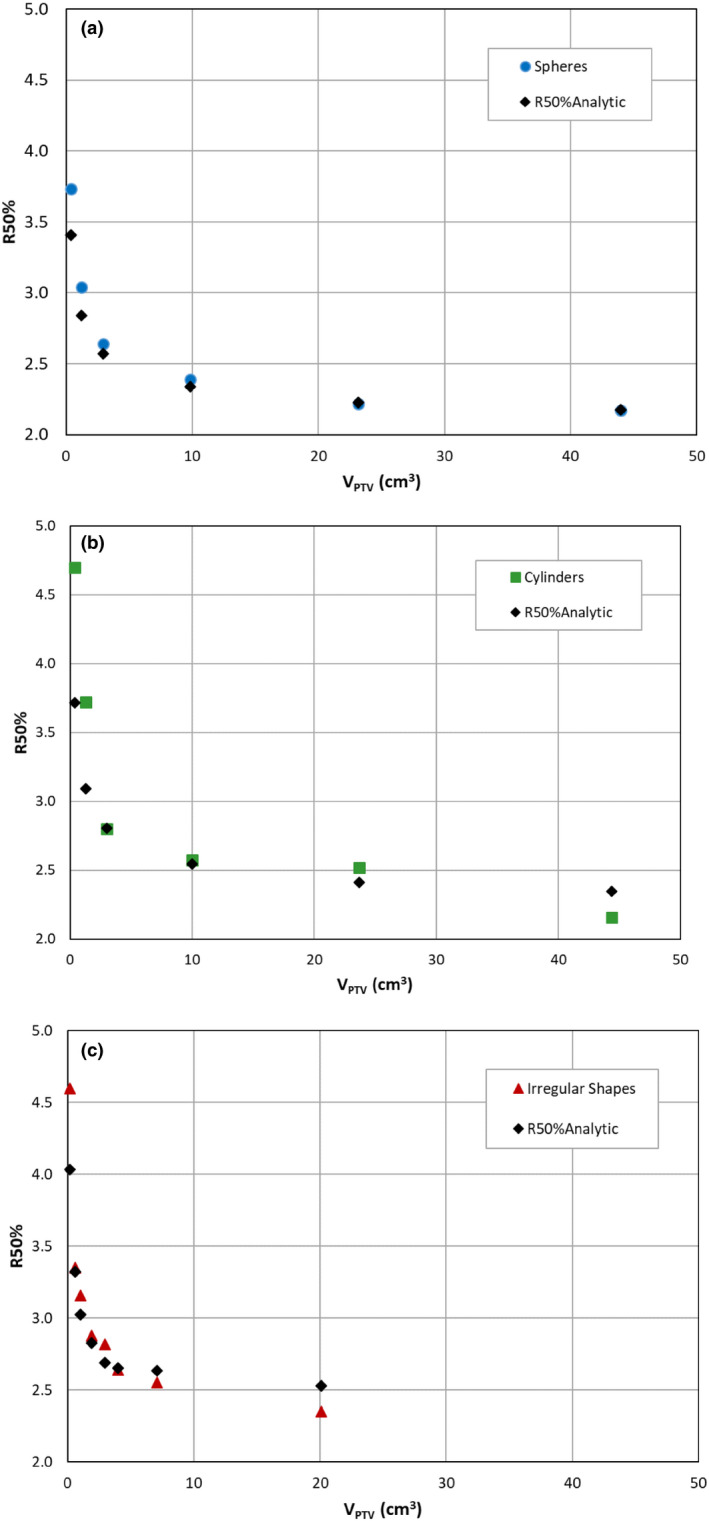
Comparison of plan R50% and R50%_Analytic_ values for (a) spherical shapes, (b) cylindrical shapes, and (c) irregular shapes.

Treatment planning results for R50% and predicted R50%_Analytic_ values are summarized in Table 3 and Figs. 4(a)–4(c) for the full SRS phantom study. The mean(sd) CI values 1.06(0.07) obtained from planning on these volumes indicate a high degree of conformality to be expected in SRS. The agreement between the R50% values obtained from treatment planning and those predicted by Eq. (1) is quite good overall. Agreement is better for the intermediate and large PTV volumes but worse for the very small volume PTVs.

**FIG. 5:**
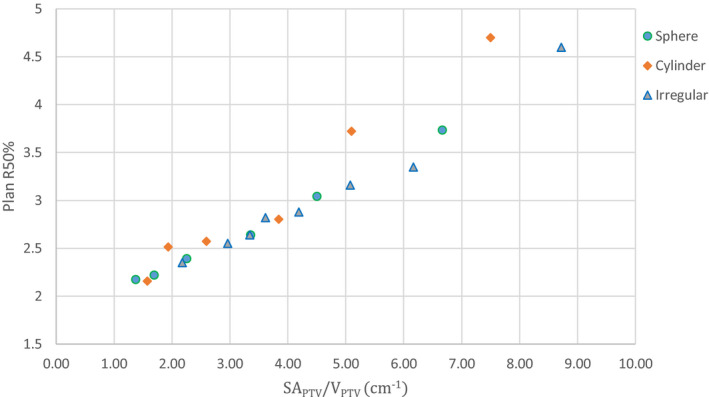
Results of SRS PTV phantom study showing the relationship of plan R50% vs the SA_PTV_/V_PTV_ ratio. Note the significant degree of correlation in these data as suggested by Eq. (1).

Figure 5 displays plan R50% values for all 20 PTVs as compared to the SA_PTV_/V_PTV_ ratio. As predicted by Eq. (1), a dominantly linear correlation of R50% with the SA_PTV_/V_PTV_ ratio can be observed.

## 4 DISCUSSION

Our goal in this research is to understand the relationship between R50% and SA_PTV_ and demonstrate a physically reasonable explanation for this dependence. The planning studies provide the direct demonstration of the surface area dependence, while the R50%_Analytic_ given in Eq. (1) provides a physically reasonable and clinically useful methodology for predicting achievable values for the treatment planning metric R50%. The good agreement seen between the actual R50% treatment planning results and those values predicted by R50%_Analytic_ in Eq. (1) satisfies this objective for the range 0.6 cm^3^ < V_PTV_ < 44 cm^3^. We call this observed dependence of R50% on SA_PTV_ the surface area effect. This appears to be a phenomenon not previously elucidated clearly in the literature of SRS, but it has been carefully explored in our previous work in lung SBRT.^12^, ^13^

R50% dependencies in Eq. (1), as indicated by the term $\frac{SA_{\mathrm{PTV}}}{V_{\mathrm{PTV}}}\Delta r\left\{1+\left[\frac{\Delta r}{r_{\mathrm{PTV}}}\right]+\frac{1}{3}{\left[\frac{\Delta r}{r_{\mathrm{PTV}}}\right]}^{2}\right\}$, include the PTV size characteristics SA_PTV_/V_PTV_ ratio, the effective PTV radius *r_PTV_*, and a dose fall‐off parameter Δr. It is expected that Δr is dependent on the photon energy and delivery technology and has been characterized for our proposed delivery scheme as illustrated in Fig. 2 and Eq. (5). Equation (2) shows the relationship of *r_PTV_* to V_PTV_ assuming the PTV is spherical. Both of these parameters (∆r and *r_PTV_*) vary more slowly than the SA_PTV_/V_PTV_ ratio over the range of PTVs sizes studied. The SA_PTV_/V_PTV_ ratio ranges approximately from a minimum of 2 cm^−1^ to a maximum of 8 cm^−1^ and this factor of 4 change is the dominant influence on the behavior of R50%_Analytic_ as the PTV size changes.

To isolate more specifically the role of SA_PTV_, a limited study of 4 cm^3^ nominal volume PTVs of different shapes consisting of one sphere and four cylinders of varying elongation was conducted. Because Δr and *r_PTV_* that are parameterized only on the basis of V_PTV_ in the R50%_Analytic_ of Eq. (1), ∆r and V_PTV_ would remain constant in R50%_Analytic_ and the only variable is SA_PTV_. The dependence of R50% on SA_PTV_ is clearly demonstrated in Fig. 3. The cylindrical shapes ranged from “pencil‐like” to “coin‐like” and indicates the R50% dependence is truly related to SA_PTV_ and not some artifact of the orientation or aspect ratio of the cylindrical PTVs. Considering that the PTV surface represents the interface between the target volume and the normal tissue, it should not be surprising that the SA_PTV_ has a marked effect on the amount of normal tissue subjected to the intermediate dose spill as quantified by R50%. For SRS, brain is the primary normal tissue of interest and, therefore, SA_PTV_ serves as the basis for the normal brain tissue volume susceptible to high doses. Given the same degree of conformality, a larger PTV surface area in a cranial SRS plan means more healthy brain tissue is exposed to the highest dose region. That exposure would propagate out to all isodose clouds outside the target including the 50% isodose cloud defining the R50%. Furthermore, the surface area effect would be a characteristic of any treatment modality capable of high conformality since the SA_PTV_ is a property of the PTV and not the radiation delivery technology. We note similarity of this PTV surface area study to a statement in the publication of Goldbaum et al.^10^ In that work, the authors hypothesized that for a cohort of PTVs with nearly equal volumes “an increase in TV12 could be related to an increase in the surface area of the target” (note TV12 is the equivalent V12 Gy). Goldbaum and co‐workers attempted to use effective ellipsoids to quantify PTV surface area but the approached proved ineffective at improving results. Yet as we see from this work, the PTV surface area effect is an important factor in predicting the plan R50%.

A more general study was conducted that evaluated 20 PTVs covering a range of characteristics with relevance to SRS. These PTVs consisted of six spheres, six cylinders, and eight irregular shapes with volumes from 0.18 to 44.38 cm^3^ and surface areas ranging from 1.57 to 69.77 cm^2^ as summarized in Table 3. The results from this study are observed in Figs. 4(a)–4(c) and 5. Figure 4 displays the behavior of R50% as a function of the PTV size expressed as V_PTV_ for spherical [Fig. 4(a)], cylindrical [Fig. 4(b)], and irregular [Fig. 4(c)] shaped structures. The sharp increase we observe in R50% for V_PTV_ < 2 cm^3^ is similar to clinical results published elsewhere for highly conformal treatments.^1^, ^6^, ^9^, ^11^, ^18^, ^19^ Also shown on Figs. 4(a)–4(c) are the R50%_Analytic_ predictions from Eq. (1). Very good agreement of the R50%_Analytic_ predictions with actual achieved R50% values is seen, especially for the intermediate size PTVs. Mean(sd) values for the Plan R50%/R50%_Analytic_ ratio are 1.04(0.08) as obtained from data in Table 3. Differences between plan R50% and R50%_Analytic_ are significantly larger for the smaller PTV volumes. Reasons for the larger discrepancy at small PTV volumes are not fully understood. However, contributing factors may stem from the discretization of the CT image set where voxelation contributes to the uncertainties associated with PTV characteristics such as SA_PTV_ or V_PTV_. For example, a 0.2 cm^3^ spherical PTV volume would have a radius of 0.36 cm. Given the voxel dimensions of the CT data set 0.07 × 0.07 × 0.1 cm, the edge of a voxel would be 20‐25% of the radius and errors of a few voxels could be significant. The dose calculation accuracy may also be affected by small PTV volumes. In our case, a 0.1 × 0.1 × 0.1 cm calculation grid matrix could result in interpolation uncertainties. Zhao et al.^1^ suggested that, for small PTV volumes, dose drop‐off is extremely sensitive to location, target shape, and beam settings and discussed the limitation of treatment planning systems to accurately compute dose for small targets. The dose distribution, the V_PTV_, and the SA_PTV_ accuracies are all compromised by the voxelation of the CT and other details of the treatment planning system; these inaccuracies increase for small PTVs. Clearly, one should exercise caution when interpreting R50% values for small PTV volumes.

Also noteworthy is the high degree of correlation of the planning R50% values with the SA_PTV_/V_PTV_ ratio as illustrated in Fig. 5. While the dependence on SA_PTV_ and V_PTV_ in Eq. (1) is complex, the dominant influence of the linear SA_PTV_/V_PTV_ ratio factor is obvious in Fig. 5 and is further evidence of the importance of the surface area effect in highly conformal treatment techniques.

While the main focus of this work was to elucidate the potential effect of SA_PTV_ on intermediate dose spill as measured by R50%, the R50%_Analytic_ prediction from Eq. (1) does require the input of the parameter Δr which is the estimated distance from the PTV surface to the V_IDC50%_ surface. Based on a study of spherical PTV volumes, estimates of Δr were obtained for our proposed treatment technique as displayed in Table 2, Fig. 2, and Eq. (5). There are published data on GM values achieved in cone‐based SRS. For example, Bova et al.^20^ suggest that for 2‐cm circular collimated beams, a GM of 0.3 cm is achievable using a 6 MV linac photon beam. Interestingly, 0.3 cm is the Δr for a 1.3 cm diameter spherical PTV treated with 6MV FFF VMAT on an HD MLC as determined by Eq. (5). Realistically, the effective aperture of the MLC when treating a 1.3‐cm diameter sphere is likely close to 2cm much of the time. Thus, the result for GM determined in this work corresponds well to the clinically achieved GM of Bova et al. This lends credence to the ∆r values obtained from Eq. (5) used in our R50%_Analytic_ model. A more comprehensive evaluation of Δr under more diverse treatment delivery conditions is a topic requiring further study.

A clear clinical application of this work is the prediction of the R50% achievable for given SRS plan. Knowing the R50%_Analytic_ prediction would allow the planner to confidently push the optimization toward that value and gives the planner a better idea of the actual quality of the final plan. While this work provides such a prediction methodology, it does so in simplified approach. Centrally located PTV volumes without additional normal tissue constraints is a much easier optimization problem than would typically exist in realistic clinical SRS cases. The cranium encompasses a large mass of normal brain tissue as well as many critical structures such as the brainstem and optic chiasm. When one considers the PTV location as well as proximal normal tissues, optimization would be more constrained and conformality compromised. The planning outcomes in these more difficult circumstances could be different from those obtained in this study. Nonspherical and asymmetric V_IDC50%_ volumes are likely and R50% values obtained may underperform those predicted by Eq. (1). Yet, based on application of the conservation of integral dose hypothesized by Reese et al.,^21^ the V_IDC50%_ and thus R50% may not dramatically change volume even if the shape is very asymmetric and thus R50%_Analytic_ may still be reasonable. Thus, we do believe the R50%_Analytic_ prediction has value in guiding the planner in searching for acceptable intermediate dose spill indexed by R50%.

It is evident from this work that the surface area of the PTV is a significant factor in determining the R50% achievable for any clinical SRS plan. It would be advantageous to know this value in all clinical situations. At present we are aware of no commercially available RTPS that reports the surface area as a component of structure properties. We developed a script that extracted the structure surface area using the information contained in the 3D surface mesh representation. The script provided SA_PTV_ values that proved predictive of the R50% for irregular structures for which analytical means for obtaining surface area are not available. If SA_PTV_ was a standard reported structure property in all RTPSs, it could facilitate wider investigations of the surface area effect on R50% or other intermediate dose spill metrics by other researchers.

## 5 CONCLUSIONS

This research has demonstrated a strong relationship between R50% and SA_PTV_ in SRS treatments that we call the surface area effect. The surface area effect has not been fully appreciated in previous research in highly conformal treatment techniques. Eq. (1) clearly establishes a physically reasonable, quantitative relationship between R50% and SA_PTV_. The R50%_Analytic_ prediction obtained from Eq. (1) establishes an excellent quantitative theoretical approximation of R50% in linac‐based, 6 MVFFF, MLC collimated SRS and provides useful guidance in treatment planning to reduce the intermediate dose spill.

## DATA VALUE STATEMENT

The data that support the findings of this study are available from the corresponding author upon reasonable request.
